# Exerting the Appropriate Application of Methylprednisolone in Acute Spinal Cord Injury Based on Time Course Transcriptomics Analysis

**DOI:** 10.3390/ijms222313024

**Published:** 2021-12-01

**Authors:** Liang-Yo Yang, Meng-Yu Tsai, Shu-Hui Juan, Shwu-Fen Chang, Chang-Tze Ricky Yu, Jung-Chun Lin, Kory R. Johnson, Hendrick Gao-Min Lim, Yang C. Fann, Yuan-Chii Gladys Lee

**Affiliations:** 1 Department of Physiology, School of Medicine, China Medical University, Taichung 404, Taiwan; yangly@mail.cmu.edu.tw; 2 Graduate Institute of Biomedical Informatics, College of Medical Science and Technology, Taipei Medical University, Taipei 110, Taiwan; meju17@gmail.com (M.-Y.T.); hendrick.san@gmail.com (H.G.-M.L.); 3 Department of Physiology, School of Medicine, Taipei Medical University, Taipei 110, Taiwan; juansh@tmu.edu.tw; 4 Graduate Institute of Medical Science, College of Medicine, Taipei Medical University, Taipei 110, Taiwan; cmbsfc21@tmu.edu.tw; 5 Department of Applied Chemistry, National Chi Nan University, Nantou County 545, Taiwan; ctyu@ncnu.edu.tw; 6 School of Medical Laboratory and Biotechnology, College of Medical Science and Technology, Taipei Medical University, Taipei 110, Taiwan; lin2511@tmu.edu.tw; 7 Intramural IT and Bioinformatics Program, Division of Intramural, National Institute of Neurological Disorders and Stroke, National Institutes of Health, Bethesda, MD 20892, USA; johnsonko@mail.nih.gov (K.R.J.); fann@ninds.nih.gov (Y.C.F.)

**Keywords:** spinal cord injury, methylprednisolone, time course transcriptomics, inflammation, glycolysis, oxidative stress, neurological system

## Abstract

Methylprednisolone (MP) is an anti-inflammatory drug approved for the treatment of acute spinal cord injuries (SCIs). However, MP administration for SCIs has become a controversial issue while the molecular effects of MP remain unexplored to date. Therefore, delineating the benefits and side effects of MP and determining what MP cannot cure in SCIs at the molecular level are urgent issues. Here, genomic profiles of the spinal cord in rats with and without injury insults, and those with and without MP treatment, were generated at 0, 2, 4, 6, 8, 12, 24, and 48 h post-injury. A comprehensive analysis was applied to obtain three distinct classes: side effect of MP (SEMP), competence of MP (CPMP), and incapability of MP (ICMP). Functional analysis using these genes suggested that MP exerts its greatest effect at 8~12 h, and the CPMP was reflected in the immune response, while SEMP suggested aspects of metabolism, such as glycolysis, and ICMP was on neurological system processes in acute SCIs. For the first time, we are able to precisely reveal responsive functions of MP in SCIs at the molecular level and provide useful solutions to avoid complications of MP in SCIs before better therapeutic drugs are available.

## 1. Introduction

Spinal cord injuries (SCIs) can arise due to direct or indirect insults. Regardless of the insult type, anti-inflammatory treatment is recommended in the shortest possible time after an SCI to minimize complete or partial damage due to swelling [1]. Drugs approved for treatment of inflammation include those members of the glucocorticoid class (e.g., dexamethasone). Of these, methylprednisolone (MP) is the only drug reported to be effective for acute SCI if administered within 8 h (h) of insult [2].

However, MP has been shown to induce a wide range of side effects, and there has been a long debate over MP usage for SCIs. The National Acute Spinal Cord Injury Study (NASCIS) I-III trial was an initial supporter of beginning routine use of MP in acute SCI conditions [3], as significant motor improvement was seen when comparing high-dose MP to a placebo. Then, a literature review and guideline article criticized the use of MP for SCIs [4] because it caused complications like wound infections, gastrointestinal hemorrhage, and thrombophlebitis, which were more common in the MP-treated group. Taken together, one could have concluded that some patients experience substantial benefits from MP, while others develop serious complications. To settle this medical case management debate, the beneficial and adverse aspects of MP with specific molecular scopes and pathways should exactly be determined. To date, the molecular effects of MP have remained unexplored. Applications of genomics to better understand responses to SCIs have been successful in enabling the discovery of many genes involved in repair, regeneration, and pathophysiology [5,6,7,8,9,10,11]. However, no systematic profiling has been conducted on MP’s effects in an intensive time-course fashion. The primary objective of this preliminary study is to provide a scientific base for the management of spinal cord injury that improves outcomes for patients by delineating the efficacy and side effects of MP on SCIs, and to determine what MP cannot achieve with SCIs at the molecular level using a transcriptomics data analysis. In this way, we should be able to distinguish the population of patients who might benefit from MP from those who might develop complications.

To achieve our goals, we conducted a biologically duplicated SCI rat experiment with and without injury and with and without MP administration, and assessed results at 0, 2, 4, 6, 8, 12, and 24 h after the injury. A gene expression microarray was used to examine results of lesions to spinal cords. In addition, we combined differential gene expressions and a Venn diagram to obtain three distinct gene classes of (1) side effect of MP (SEMP), which comprised genes that were not changed by the SCI but were altered after MP treatment, (2) competence of MP (CPMP), which comprised genes that were changed by the SCI and recovered after MP treatment, and (3) incapability of MP (ICMP), which included genes that were changed by the SCI and continued changing after MP was administered. Based on these results, we may be able to specifically tackle the side effects of MP by avoiding populations of patients who might develop complications, or devise other measures to improve the condition.

## 2. Materials and Methods

### 2.1. Spinal Contusions and Grouping

Sixty female Long Evans rats (8~9 weeks old) were purchased from the National Laboratory Animal Center (Taipei, Taiwan). All animals were housed in a 12 h light/dark cycle and had free access to water and food at all times. For animal care and surgical procedures, we followed the National Institutes of Health (NIH) Guide for the Care and Use of Laboratory Animals (NIH Publications No. 80–23, revised 1978) and all surgical protocols were approved by the Institutional Animal Care and Use Committee of Taipei Medical University Laboratory Animal Center.

The SCI model in rats has been described in detail elsewhere [12,13,14,15]. For all surgeries, animals were anesthetized with a mixture of Zoletil 50 (25 mg/kg) (Virbac Laboratories, Carros, France) and Rompun (Xylazine, 10 mg/kg, Bayer, Leverkusen, Germany) via intraperitoneal injections. Following a T9~T10 laminectomy, SCI was induced using a New York University Impactor by dropping a 10 g weight from a height of 25 mm. Perioperative care was based on NASCIS guidelines, as described in a previous publication [16].

The solubility of MP in water is very low (0.024 mg/mL). Therefore, MP (30 mg/kg) was dissolved in 0.5 mL 70% ethanol, which was prepared using sterilized Milli-Q^®^ water (Merck MilliporeSigma, Burlington, MA, USA) and 100% ethanol. MP or non-MP control (0.5 mL 70% ethanol) was administered intravenously 15 min (min) after SCI injury during a 25 min period. In the MP experiment, samples were designed in duplicate, and 60 animals were randomly assigned to four groups: (1) SCI with 70% ethanol (SCI-EtOH), for injured rat without MP administration; (2) SCI with MP (30 mg/kg) (SCI-MP), for injured rat with MP administration; (3) sham-EtOH (S-EtOH), for non-injured rat without MP administration, and (4) sham-MP (S-MP), for non-injured rat with MP administration. Animals were sacrificed at 2, 4, 6, 8, 12, 24, and 48 h after being medicated. Additional animals including S-EtOH and SCI-EtOH were sacrificed immediately as the 0 h control. The 5 mm lesioned spinal cord was removed, immediately frozen in liquid nitrogen, and stored in liquid nitrogen prior to ribonucleic acid (RNA) extraction.

### 2.2. RNA Extraction and Microarray Procedures

Total RNA was extracted by the TRIzol method [17], and the integrity of the RNA was examined by an Agilent Bioanalyzer (Agilent Technologies, Santa Clara, CA, USA) before oligo-microarray procedures. The chip used for this spinal cord injury project was the GeneChip^®^ Rat Gene 1.0 ST Array (Affymetrix, Santa Clara, CA, USA). This gene array differs from traditional 3′-end expression arrays, such as Rat Expression Set 230 or Rat U34 array. The design of the Rat Gene 1.0 ST array contains 26 probes across the full length of the gene and in total contains 27,342 genes. Sample labeling and hybridization were processed according to the manufacturer’s instructions in the Microarray Core Facility of the National Human Genome Research Institute-National Institutes of Health (NHGRI-NIH, Bethesda, MD, USA). Then the arrays were scanned on an Affymetrix GeneChip scanner.

### 2.3. Data Preprocessing

#### 2.3.1. Data Normalization

In the first step of the protocol, our microarray data were normalized using the robust multi-array average summarization algorithm [18] in probe sets. We processed data on a per-chip and per-gene normalization basis using GeneSpring GX 11 software (Agilent Technologies). Our data were finally transformed into a log2 format.

#### 2.3.2. Outlier Sample Removal

We calculated correlation values between each sample with the pair-wise Pearson method for 60 samples in our experiment. Outliers were defined as samples that exceeded 90% of the pair-wise correlation values of at least < (mean − 2 × standard deviation (SD)). We also performed a principal component analysis (PCA) to look for outlier samples falling distal to the dataset at large.

#### 2.3.3. Noisy Gene Detection

We filtered noisy genes by the error in evaluating the coefficient of variation (CV) of duplicated samples in our experiments. The standard of excluding noisy genes should be objective; otherwise, one could lose too many probe sets and affect biological variations in our experiments. Therefore, probe sets with at least 16 pairs of duplicate samples that had a CV of <50 were retained for further analysis. After the noise analysis was performed, the dataset was reduced from 27,342 to 21,653 probe sets.

### 2.4. Experimental Design and Statistical Analyses

Four steps were used to determine the three gene classes of MP-responsive genes (SEMP, CPMP, and ICMP) from the original experimental design grouping.

Step 1. Group definitions.

Four groups were designed in the MP experiment—S-EtOH, S-MP, SCI-EtOH, and SCI-MP with three variables of injury (SCI), medication (MP administration), and time as shown in Table 1.

Step 2. Differentially expressed gene (DEG) sets.

**Figure 1:**
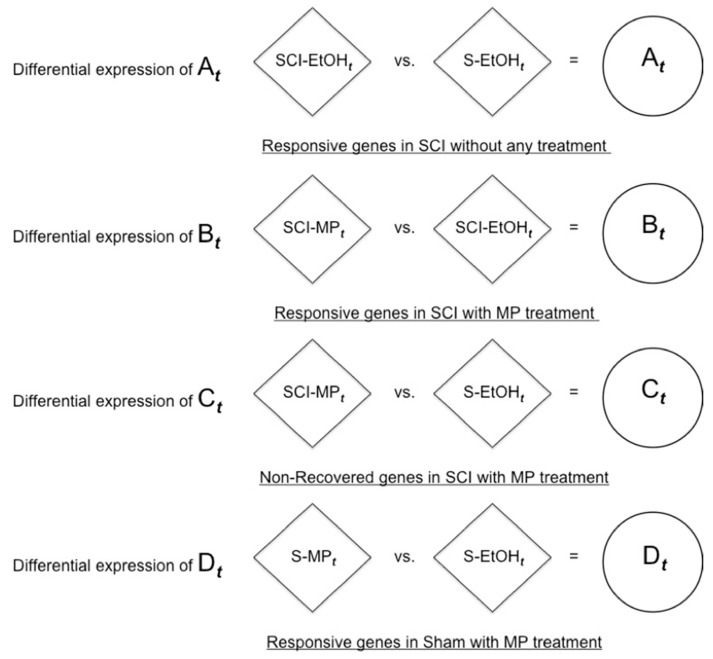
Four differentially expressed gene sets.

We obtained four differential expression gene sets of A*_t_*, B*_t_*, C*_t_*, and D*_t_* by pair-wise comparisons of the four groups at *t* hours (*t* = 2, 4, 6, 8, 12, 24, 48 h) through a statistical analysis as shown in Figure 1 and defined as follows:

- A*_t_*: responsive genes in SCI with no treatment, SCI-EtOH*_t_* versus (vs.) S-EtOH*_t_*.
- B*_t_*: responsive genes in SCI with MP treatment, SCI-MP*_t_* vs. SCI-EtOH*_t_*.
- C*_t_*: non-recovered genes in SCI with MP treatment, SCI-MP*_t_* vs. S-EtOH*_t_*.
- D*_t_*: responsive genes in the sham group with MP treatment, S-MP*_t_* vs. S-EtOH*_t_*.

In this experiment, we used GeneSpring GX 11 to perform statistical analysis based on a one-way analysis of variance (ANOVA) for multiple comparisons using the Benjamini and Hochberg false discovery rate (FDR) [19] with a cutoff *p* = 0.05 among the four groups at each time point (0, 2, 4, 6, 8, 12, 24, and 48 h post-injury). Then, we used Tukey’s honest significant difference (HSD) for pair-wise comparisons of groups. A *p* value of differential expression between two groups of ≤0.05 was defined as statistical significance, otherwise it was not significant.

**Figure 2:**
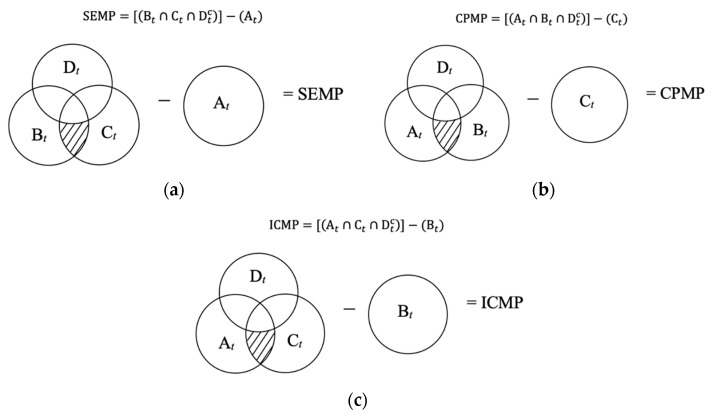
Venn diagram visualization of (**a**) side effect of MP (SEMP), (**b**) competence of MP (CPMP), and (**c**) incapability of MP (ICMP) gene classes.

**Figure 3:**
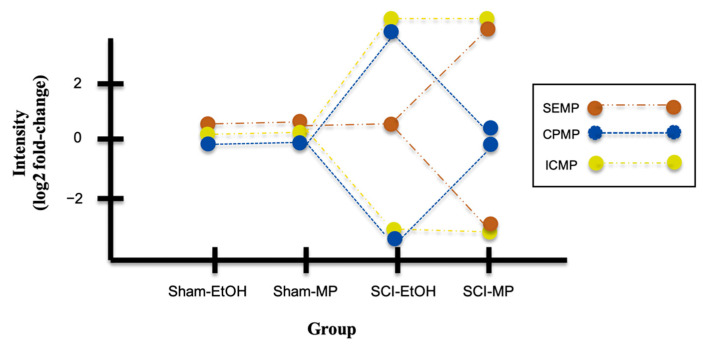
Expression profiles of three gene classes in four groups of MP experiment.

Step 3. Venn diagram to obtain gene classes.

(3a) First, we used this following general formula to define gene class.

(1) $$\mathrm{Gene}\;\mathrm{class}=[\left[X_{t}\cap Y_{t}\cap D_{t}^{c}\right]-Z_{t}],$$

where $X_{t\;},Y_{t},{\;Z}_{t}$ can be $A_{t},B_{t},{\;C}_{t}$ interchangeably, but not at the same time, *t* = 2, 4, 6, 8, 12, 24, 48 h. $D_{t}^{c}$ is the complement of the differentially expressed $D_{t}$ gene set. Therefore, a Venn diagram was created with four differential expression gene sets as demonstrated in Figure 2 to obtain three gene classes that reflect MP’s responses—SEMP, CPMP, and ICMP at *t* hours (*t* = 2, 4, 6, 8, 12, 24, 48 h).

(3b) The hypothetical expression profiles with log2 fold-change are used to show symmetric measure, when the change decreases by an equivalent amount, e.g., greater than zero is over-expression, and less than zero is down-regulation. The profiles are displayed with certain colors: brown for SEMP, blue for CPMP, and yellow for ICMP, as shown in Figure 3. Meanwhile, these three chosen gene classes with or without statistical significance (*p* ≤ 0.05) in the DEG sets (A*_t_*, B*_t_*, C*_t_*, and D*_t_*) are indicated in Table 2.

Step 4. Functional analysis.

We used the tool FatiGO (Babelomics 3, http://babelomics.bioinfo.cipf.es accessed on 26 June 2011) [20,21] to conduct a gene ontology (GO) analysis. In FatiGO, two groups of genes of interest can be tested simultaneously by means of Fisher’s exact test, and all *p* values were adjusted by the FDR. FatiGO was implemented in the nested inclusive analysis, where the test is performed recursively until the deepest level is achieved where significance is obtained, and only this last level is reported. Therefore, GO terms were automatically tested from levels 3 to 9 and only the deepest significant term was reported for each branch in the directed acyclic graph (DAG).

## 3. Results

### 3.1. Functional Enrichment Analysis

The first set of questions was aimed at identifying MP-responsive genes. Through the three steps described in Section 2.4 we were able to obtain three distinct gene classes: (i) SEMP comprised genes that did not change after injury but were altered post-treatment, (ii) CPMP included genes that recovered post-treatment, and (iii) ICMP comprised genes that sustained change after injury and post-treatment.

Significant GO results were obtained by comparing three classes of genes (SEMP, CPMP, and ICMP) with rest of genome (ROG) via the FatiGO tool. We arranged the time points into four phases in our comparative analysis. Pair-wise comparisons included SEMP vs. ROG, CPMP vs. ROG, and ICMP vs. ROG at each phase: 2~6, 8, 12, and 24~48 h. The number of genes and associated biological process (BP) terms with statistical significance (*p* ≤ 0.05) of each class at different time phases are given in Table 3 and Table 4, respectively. The detailed list of genes and their associated BP terms with their significance *p* value (*p* ≤ 0.05) shown in DAG are provided in Supplementary Files S1 and S2, respectively. The most representative and significant terms of GO BPs and cellular components (CCs) are listed in Table 5. Note that in Table 3 and Table 4, higher gene numbers did not necessarily produce more BP terms. A significant BP term required a sufficient number of functionally related genes distributed in a single BP cluster.

**SEMP vs. ROG.** We found that SEMP mainly responded at 2~6 and 24~48 h, while it was not significant at 8 or 12 h. SEMP was related to the functions of transport, metabolic processes, energy mechanisms, and liver development in the mitochondrion component at 2~6 h. Although there were no significant responses in the two middle phases, SEMP was affected at 24~48 h in regulation of cell differentiation, cell cycle, cell proliferation, phosphorylation, metabolic processes, deoxyribonucleic acid (DNA) geometric changes, translation, and chemotaxis in the component of ribosomes, neuron projections, and cell fractions (see details in Table 5, SEMP part).

**CPMP vs. ROG.** We found that CPMP mainly responded at 8 and 12 h, while it was not significant early at 2~6 h or late at 24~48 h. At 8 h, CPMP was associated with multiple aspects: immune processes, wound healing processes, chemotaxis (cell migration), transcription factor activities, angiogenesis, apoptosis (programmed cell death), integrin-mediated signaling pathways, cortical actin cytoskeletal organization, cell adhesion, and hormone secretion (secretion by cells) in the components of the extracellular space and cell surface. Responsive functions of CPMP at 12 h were only ‘de novo’ posttranslational protein folding in the component of the endoplasmic reticulum (see details in Table 5, CPMP part).

**ICMP vs. ROG.** Most of the ICMP-responsive functions through time phases were mainly correlated with ion transport, neurotransmitter transport/secretion (secretion by cells), neuron differentiation, transmission of nerve impulses, and metabolic processes except at 8 h. We observed that what occurred at 8 h was associated with responses to reactive oxygen species (ROS), DNA ligation during DNA repair, transcription from RNA polymerase II promoters, DNA replication, cell proliferation, and aging (see details in Table 5, ICMP part).

### 3.2. Interactive Time Phase Visualization

**Figure 4:**
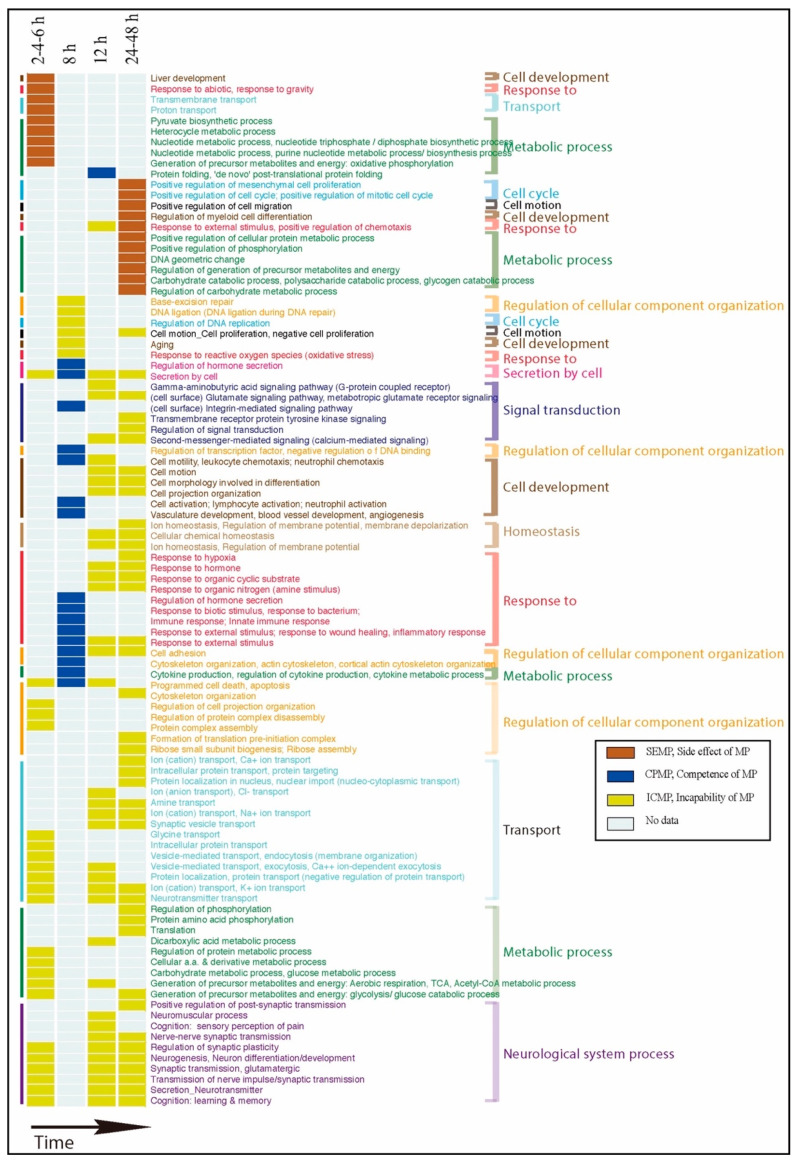
Visualization of the associated biological process of three gene classes in a time series fashion. The colors of each gene class are as indicated.

The above functional BPs of SEMP, CPMP, and ICMP were visualized in different colors in a time-phase fashion, as shown in Figure 4. Functions of SEMP and ICMP were dominant at the early time of 2~6 h; however, some functions had been restrained by CPMP at 8 and 12 h. This might have been due to the prime efficacy of MP at 8~12 h, while SEMP and ICMP were again dominant at 24~48 h. Furthermore, one can clearly see how SEMP, CPMP, and ICMP interacted in a time course when MP was administrated for an acute SCI. When MP exerted its greatest efficacy at 8 and 12 h, CPMP demonstrated its protective effect, whereas SEMP and ICMP were inhibited during that period of time. Before and after the periods that MP efficacy had not begun or had decreased, respectively, the functions associated with side effects (SEMP) and what MP could not treat (ICMP) came into play.

## 4. Discussion

We know that MP treatment lowers inflammation but induces side effects that have been clinically proven. The focus of our research was to identify and specify the molecular effects of MP treatment on SCIs and provide potential candidates to (1) determine how and when MP treatment is beneficial; (2) help select patients who might benefit from MP and avoid those who might develop complications using MP; and (3) provide directions for further alternate therapeutic targeting in the near future and clinically approved dietary supplementation when MP is used for SCIs. To accomplish our objectives, we investigated the functions of MP-responsive genes and identified three distinct classes of genes: CPMP, SEMP, and ICMP, which would provide clues for these goals.

### 4.1. The Beneficial Molecular Mechanism of MP Usage: MP Protects Nerves by Reducing Inflammation

NASCIS I, II, and III supported the use of MP and demonstrated motor function improvement [3,22]. The glucocorticoid, MP, mainly suppresses this inflammatory event, since inflammatory cells release neurotoxins, such as proinflammatory cytokines and chemokines, free radicals, excitotoxic amino acids, and nitric oxide, all of which can create axonal and neuronal deficits. Therefore, lowering inflammation is equivalent to protecting neuronal cells from mass attack by neurotoxins at the same time. Microglia, neuroglia located in the brain and spinal cord, are such types of macrophage cells, which act as the main form of active immune defense in the central nervous system (CNS). Activated microglia induce reactive astrocytes, which cause loss of the ability to promote neuronal survival, outgrowth, synaptogenesis, and phagocytosis, and induce the death of neurons and oligodendrocytes [23]. Therefore, MP use in the first place prevents activation of microglia and is also neuroprotective.

In our study we demonstrated that the significant functions from good responsive genes to MP administration at 8 h were inflammatory responses, blood coagulation (wound healing), leukocyte/neutrophil chemotaxis, cytokine production, negative regulation of DNA binding, angiogenesis, cortical actin cytoskeletal organization, cell adhesion, regulation of hormone secretion, etc., which correspond with most observations of secondary damage due to SCIs [24].

Another point worth mentioning is that in our study we showed evidence that the beneficial temporal change occurred in 8 h (and some in 12 h) when MP was administered on the SCI. That means that 8 h is critical for acute injury as inflammatory events take action and cause unmendable neuronal deficits and destruction. This phenomenon was also observed in clinical trials. The investigators of NASCIS II concluded that neurological recovery was improved in patients who received MP within 8 h of injury and that the treatment was relatively safe, with similar mortality and morbidity between the treatment groups [2]. In NASCIS III, it was also shown that 24 h of MP initiated within 8 h was to be effective and safe. Altogether, it seems that if an appropriate dose of MP is used within 8 h of injury either in humans or animals, it protects neuronal cells from mass attack by neurotoxins, such as cytokines and chemokines, and promises a better outcome.

### 4.2. Mechanisms of the Side Effects Induced by MP Administration: Metabolic Problems Leading from Glycolysis and Oxidative Stress to the Warburg Effect

The so-called side effects of MP here by definition are biological events that remained the same after an SCI occurred but deteriorated after MP administration. This is a very likely cause of a certain population experiencing complications after MP treatment. In this study, we clearly indicated that the side effect of MP in SCI patients involved glycolysis, pyruvate biosynthetic processes, heterocyclic metabolic processes, proton transport, and liver development early at 2~6 h after MP administration.

MP is a glucocorticoid with a wide range of effects, including changes to metabolism. The use of MP can induce both glucose and insulin levels in non-diabetic SCI patients [25,26], and the higher blood sugar levels are further taken up by cells and processed as glycolytic metabolism (glycolysis). However, with excessive glycolysis but a low oxygen supply (due to damage to the vasculature of the spinal cord), oxidative phosphorylation in mitochondria cannot completely proceed, resulting in cellular acidosis (lactate acid, uric acid, oxalate, citric acid, etc.), free radicals (such as hydroxyl, nitric dioxide, etc.), and less energy than it was supposed to produce.

The oxidative stress/oxidative damage further causes neuronal cell death, axonal demyelination and severing, microglial activation, and glial scar formation [27]. This vicious cycle of glycolysis and partial oxidative phosphorylation in mitochondria after MP administration in SCIs can lead to the “Warburg effect” and a series of metabolic malfunctions. The Warburg effect might lead to oncogenesis [28]. As indicated from our data analysis of SEMPs at 24~48 h, there were “positive regulation of mitotic cell cycle”, “positive regulation of mesenchymal cell proliferation”, and “regulation of positive chemotaxis (cell migration)”, which seemed to echo cancer hallmarks.

Abnormal metabolism in the neurological system is especially serious. It depends on coordination and cooperation among neurons, astrocytes, and oligodendrocytes in the context of energy metabolism, such as glucose handling during neurodevelopment in the spinal cord and adult brain. Adenosine triphosphate (ATP) molecules are the major source of energy, as a part of glycolysis for neural progenitor cells, astrocytes, and oligodendrocytes, and their lactate products are used as resources for oxidative phosphorylation for neurons along with glucose at the same time (cell bodies and axons) [29].

### 4.3. Solution to the Side Effects of MP in SCIs

Glucose is not the only fuel source that can be used by the brain and neurological system. In fact, research has shown that ketone bodies are the preferred fuel source of the human brain, meaning that when both glucose and ketones are present, the brain will preferentially consume ketones [30].

To sum up the scenarios of the side effects and complications caused by MP, we suggest that a low-carbohydrate diet would be highly recommended when using MP treatment for non-diabetic SCI patients. Furthermore, a ketogenic diet can be considered to change the burning resources from glucose to fatty acids to reduce the Warburg effect. After all, in humans, oxidation of fats is quantitatively more important than the oxidation of glucose as a source of ATP. In this way, cellular acidosis and free radicals could be massively improved by reducing glucose intake and consequently reducing glycolytic metabolism [31].

As for patients with diabetes or hyperglycemia, clinicians should either avoid prescribing MP or use MP with special care alongside dietary restrictions, such as a ketogenic diet without carbohydrates.

### 4.4. Trauma Caused by an SCI and Inabilities to Treat it with MP

The inabilities of MP (ICMPs) to treat SCIs by definition are biological events that have been provoked (up- or downregulated) when an SCI occurs and remain the same after MP administration. Most such events are primary physical insults, dislocation or fracture of a vertebral body with subsequent spinal cord compression, and injury and ischemia of nearby glial cells and nerve cells in the spinal cord.

From our results, such ICMP events included the neurological system, neurotransmitter secretion, synaptic vesicle transport, endocytosis, exocytosis, negative protein transport, effects on ion transport, cytoskeletal organization, cell projection organization, and protein complex disassembly in the early stage of an acute SCI (at 2~6 h after MP administration), while homeostasis, cell developmental events, signal transduction, cellular component organization, etc., are involved at later stages (24~48 h after MP administration). These events occur simply because of the acute SCI and are also events that MP could not rescue or repair. Therefore, novel therapeutic strategies for lessening the SCI burden are highly needed.

In the brain and spinal cord, glial cells, including oligodendrocytes, astrocytes, and microglia, are believed to not only maintain the homeostasis of neural cells, providing ion transport and nutrients, but also have the ability to repair neural cells [32]. Particularly for oligodendrocytes, they support neurons and signal transmission in the CNS by enwrapping axons with myelin, a lipid-rich membrane structure. It was found that fatty-acid synthesis was essential to sustain adult oligodendrocyte progenitor cell-derived oligodendrocytes and efficient remyelination when focal demyelination due to spinal cord lesions occurred. Therefore, fatty acid synthesis in oligodendrocytes plays key roles in CNS myelination and remyelination [33]. Myelin is characterized by an exceptionally high lipid content (~80% of dry weight) [34]. Fatty acids are fundamental building blocks for both glycolipids and phospholipids, which comprise other large proportions of myelin membrane lipids [33].

### 4.5. Solution to the Inability Effect of MP for SCIs

Lutz and Durand [35] demonstrated that the fatty acid composition of nerve membranes (myelin and synaptosomes) was influenced by dietary oils, and dietary fatty acids can be positively involved in the control of CNS myelinogenesis [36]. Furthermore, it was indicated that omega-3 polyunsaturated fatty acids (n-3 PUFAs) might have a role in promoting remyelination after toxic injury to CNS oligodendrocytes. This might occur either via modulation of the immune system or via a direct effect on oligodendrocytes or neurons through eicosapentaenoic acid (EPA)-derived lipid metabolites [37]. The two main forms of n-3 PUFAs in the brain, docosahexaenoic acid (DHA) and EPA, inhibit oxidative stress and tumor necrosis factor (TNF)-α from primary microglia upon interferon (IFN)-γ and myelin stimulation [38]. Before a better therapeutic drug for nerve regeneration is officially developed, a suitable dietary supplement of good-quality fatty acids is highly recommended based on this empirically approved research.

## 5. Conclusions

Currently, there is no effective medication for SCIs. The only approved drug is MP, and it is suggested to be used for less than 8 h after injury to prevent further injury. However, due to the potential side effects of MP in some patients, MP is no longer recommended for routine use after an SCI. Our study is the first research that has comprehensively explored MP on SCIs at the molecular level to examine its benefits, side effects, and incapability. Therefore, this study clearly delineates the molecular genes and pathways in these three categories. Furthermore, we were able to answer why MP treatment is beneficial for 8 h after an SCI. These results will be helpful in selecting patients who might benefit from MP and those who might develop complications when using MP. With our comprehensive genome-wide data analysis approach, we have provided the basis of genes and pathways, which further provides a direction for alternate therapeutic targeting to improve the shortcomings of MP. In addition, since we have a clear idea about the mechanisms that cause adverse side effects and what MP is incapable of, clinically approved dietary supplements can be used to overcome these shortcomings for the time being.

## Figures and Tables

**Table 1:** Groups in the methylprednisolone experiment.

Variable	MP Experiment Group			
	S-EtOH	S-MP	SCI-EtOH	SCI-MP
Injury	Sham	Sham	SCI	SCI
Medication	EtOH	MP	EtOH	MP
Time (h)	0, 2, 4, 6, 8, 12, 24, 48	0, 2, 4, 6, 8, 12, 24, 48	0, 2, 4, 6, 8, 12, 24, 48	0, 2, 4, 6, 8, 12, 24, 48

MP, methylprednisolone; S, sham; EtOH, ethanol; SCI, spinal cord injury; h, hour(s).

**Table 2:** Significancy table of three gene classes against four differentially expressed gene sets.

Gene Class	A*_t_*	B*_t_*	C*_t_*	D*_t_*
	SCI-EtOH*_t_* vs. S-EtOH*_t_*	SCI-MP*_t_* vs. SCI-EtOH*_t_*	SCI-MP*_t_* vs. S-EtOH*_t_*	S-MP*_t_* vs. S-EtOH*_t_*
SEMP	×	o	o	×
CPMP	o	o	×	×
ICMP	o	×	o	×

SEMP, side effect of MP; CPMP, competence of MP; ICMP, incapability of MP. o, statistically significant (*p* ≤ 0.05); ×, not statistically significant (*p* > 0.05).

**Table 3:** Number of genes of each gene class in four different time phases.

Gene Class (vs. ROG)	Time Phase			
	2~6 h	8 h	12 h	24~48 h
SEMP	210	10	179	94
CPMP	190	232	224	182
ICMP	494	207	960	2730

ROG, rest of genome.

**Table 4:** Statistically significant (*p* ≤ 0.05) numbers of associated biological process terms of each gene class in each time phase.

Gene Class (vs. ROG)	Time Phase			
	2~6 h	8 h	12 h	24~48 h
SEMP	16	×	×	25
CPMP	×	56	3	×
ICMP	63	9	250	303

**Table 5:** List of the most representative and statistically significant gene ontology terms (biological processes and cellular components) of each gene class of SEMP, CPMP, and ICMP at different time phases.

	**SEMP vs. ROG**	
**Time Phase**	**Biological Processes**	**Cellular Components**
2~6 h	Proton transport Transmembrane transport Glycolysis Heterocyclic metabolic processes Pyruvate biosynthetic processes Purine nucleotide biosynthetic processes Liver development	Mitochondrion Organelle inner membrane Proton-transport - Two sector ATPase complex - ATP synthase complex, catalytic core F (1)
8 h	None	None
12 h	None	None
24~48 h	Regulation of myeloid cell differentiation Positive regulation of mitotic cell cycle Positive regulation of mesenchymal cell proliferation Positive regulation of phosphorylation Regulation of carbohydrate metabolic processes Positive regulation of cellular protein metabolic processes DNA geometric changes Translation Glycogen catabolic processes Positive regulation of positive chemotaxis (cell migration)	Ribosomes Neuron projections Cell fractions
	**CPMP vs. ROG**	
**Time Phase**	**Biological Processes**	**Cellular Components**
2~6 h	None	None
8 h	Defense response to Gram-positive bacteria Inflammatory response (defense response) Blood coagulation (wound healing) Leukocyte/neutrophil chemotaxis (cell migration) Cytokine production Negative regulation of DNA binding Regulation of transcription factor activity Lymphocyte/neutrophil activation Angiogenesis Apoptosis (programmed cell death) Integrin-mediated signaling pathways Cortical actin cytoskeleton organization (actin filament-based processes) Cell adhesion Regulation of hormone secretion (secretion by cells)	Extracellular space Cell surface
12 h	‘De novo’ posttranslational protein folding	Signal recognition particles, endoplasmic reticulum targeting
24~48 h	None	None
	**ICMP vs. ROG**	
**Time Phase**	**Biological Processes**	**Cellular Components**
2~6 h	Ion transport (potassium) Glycine transport Intracellular protein transport Exocytosis (calcium) Endocytosis (vesicle-mediated transport) Neurotransmitter secretion/transport (secretion by cells) Cytoskeleton organization Apoptosis (programmed cell death) Regulation of protein complex disassembly (regulation of cellular component organization) Rac protein signal transduction Regulation of cell projection organization (cell-cell signaling) Regulation of neuron differentiation (neurogenesis) Transmission of nerve impulses (synaptic transmission) Generation of precursor metabolites and energy Acetyl-CoA metabolic processes Glucose metabolic processes (hexose metabolic processes/carbohydrate metabolic processes) Regulation of phosphorylation Positive regulation of transferase activities Regulation of protein metabolic processes	Cell junctions Cell projections - Flagellin-based flagella - Neuron projections: axons and dendrites Cell fractions - Synaptosome Cell soma Vesicles - Cytoplasmic vesicles - Membrane-bound vesicles Cytosol Organelle outer membranes Golgi apparatus Cytoskeleton Membrane coat Coated pit Postsynaptic membrane
8 h	Response to reactive oxygen species DNA ligation during DNA repair (base-excision repair) Transcription from RNA polymerase II promoter Regulation of DNA replication Negative regulation of cell proliferation Aging	Perinuclear region of cytoplasm
12 h	Negative regulation of protein transport Ion transport (sodium/potassium/calcium/chloride) Amine transport Positive regulation of catalytic activities Mitochondrial transport Synaptic vesicle exocytosis (secretion by cells) Neurotransmitter secretion/transport (secretion by cells) Regulation of membrane potential Calcium-mediated signaling (second-messenger-mediated signaling) Glutamine signaling pathways Gamma-aminobutyric acid signaling pathways Apoptosis (programmed cell death) Synaptic organization Transmission of nerve impulses (synaptic transmission) Neuron differentiation (neurogenesis) Cell adhesion Cell projection organization Leukocytes/neutrophil chemotaxis (cell migration) Sensory perception of pain Response to organic cyclic substances Dicarboxylic acid metabolic processes Glycolysis (glucose catabolic process/generation of precursor metabolites and energy) Tricarboxylic acid cycle (acetyl-CoA metabolic processes) Responses to hormone stimuli Responses to organic nitrogen	Cell junctions - Intercalated discs Cell projections - Neuron projections: axons and dendrites - Ruffles - Lamellipodia - Flagellin-based flagella Cell fractions - Synaptosomes Cell soma Vesicles - Cytoplasmic vesicles - Membrane-bound vesicles Cytosol - Cytosolic ribosomes Organelle membranes Golgi apparatus Cytoskeleton - Microtubules - Actin Chloride channel complex Pre- and post-synaptic membranes Intrinsic to plasma membranes Ionotropic glutamine receptor complex Cyclic-dependent protein kinase holoenzyme complex Asymmetric synapses Growth cones Mitochondria Neuromuscular junctions Pyruvate dehydrogenase complex Tricarboxylic acid cycle enzyme complex Heterotrimeric G-protein complex
24~48 h	Ion transport (sodium/potassium/calcium) Intracellular protein transport Amine transport Synaptic vesicle exocytosis (secretion by cells) Neurotransmitter secretion/transport (secretion by cells) Negative regulation of cell proliferation Cell cycle Glutamate signaling pathway Ribosomal small subunit assembly Transmembrane receptor protein tyrosine kinase signaling pathway Second-messenger-mediated signaling Cytoskeleton organization Regulation of signal transduction Transmission of nerve impulses (synaptic transmission) Neuron differentiation (neurogenesis) Cell projection organization Cellular ion homeostasis Cell adhesion Protein amino acid phosphorylation Translation Response to hypoxia Response to organic cyclic substances Response to organic nitrogen Locomotory behavior	Cell junctions - Cell-substrate junctions Cell projections - Neuron projections: axons and dendrites - Ruffles - Filopodia - Uropod Cell fractions - Synaptosomes - Microsomes Cell soma - Perikaryon Vesicles - Cytoplasmic vesicles - Membrane-bounded vesicles Cytosol - Cytosolic ribosomes Organelle membranes Golgi apparatus Cytoskeleton - Microtubules - Actin Chloride channel complex Pre- and postsynaptic membranes Intrinsic to plasma membranes Ionotropic glutamine receptor complex Cyclic-dependent protein kinase holoenzyme complex Asymmetric synapses Growth cones Cell surfaces Perinuclear region of cytoplasm Membrane rafts Pore complex Sodium: potassium-exchanging ATPase complex cAMP-dependent protein kinase complex Sarcolemma Calcium- and calmodulin-dependent protein kinase complex Integrin complex Cleavage furrows Cell cortices Endoplasmic reticula Extracellular matrix - Proteinaceous extracellular matrix Synapse clefts

## Data Availability

The raw data from our rat model experiments are available in the National Center for Biotechnology Information, Gene Expression Omnibus (https://www.ncbi.nlm.nih.gov/geo/) with accession code of GSE15878.

## Supplementary Materials

The following are available online at https://www.mdpi.com/article/10.3390/ijms222313024/s1, Detailed information of functional enrichment analysis results are available in Supplementary Files S1 (Excel), for detailed list of genes. File S2 (PDF), for significant biological process terms of our gene classes (SEMP, CPMP, and ICMP) in each time phases (2~6, 8, 12, and 24~48 h).
