# Supplementary figures and images for: Exerting the Appropriate Application of Methylprednisolone in Acute Spinal Cord Injury Based on Time Course Transcriptomics Analysis
Source: Int J Mol Sci. 2021 Dec 1;22(23):13024. doi: 10.3390/ijms222313024 (PMC8657964; doi:10.3390/ijms222313024)

## SEMP 2-4-6h

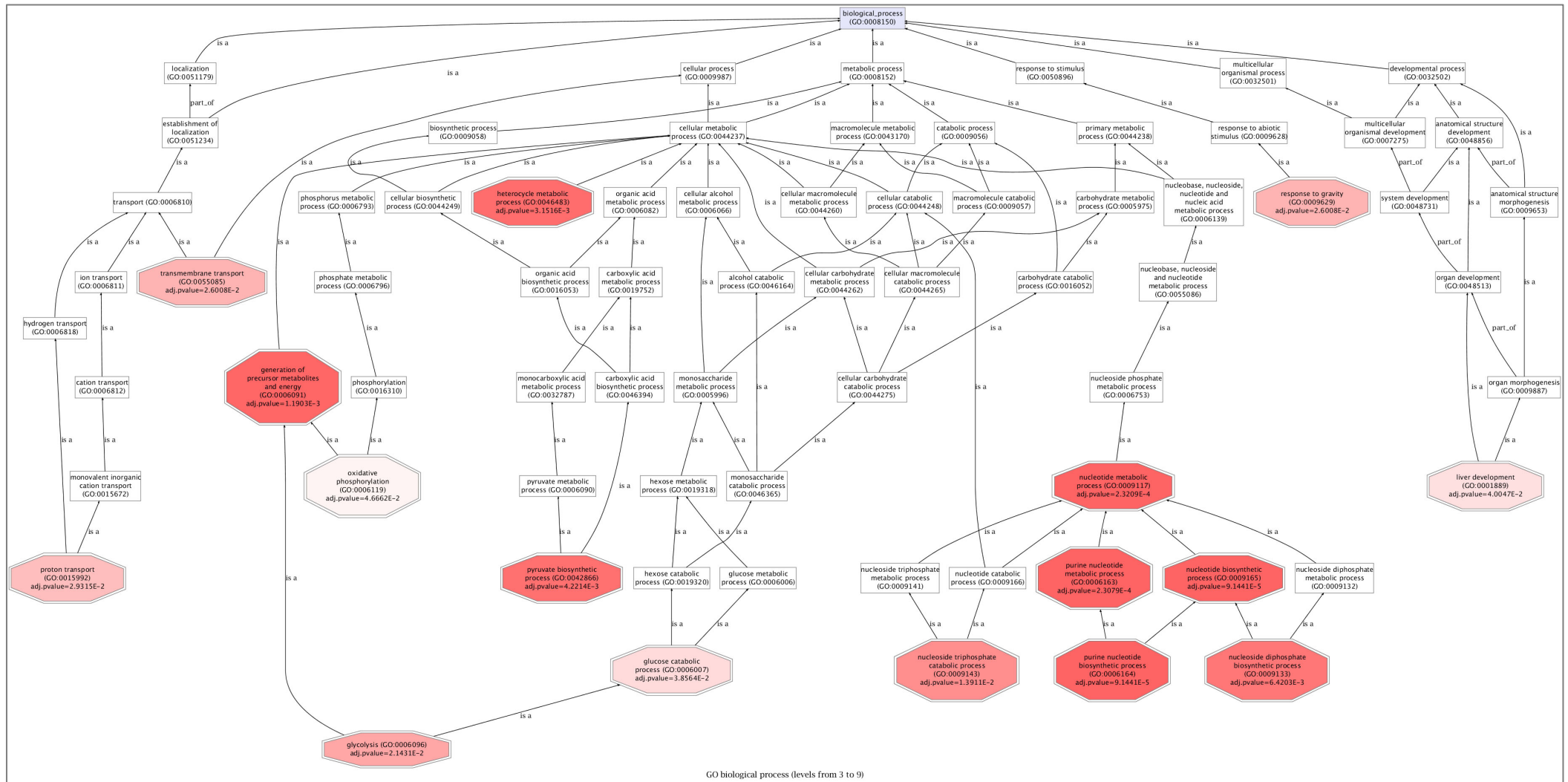

## SEMP 24-48h

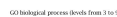

## CPMP 8h

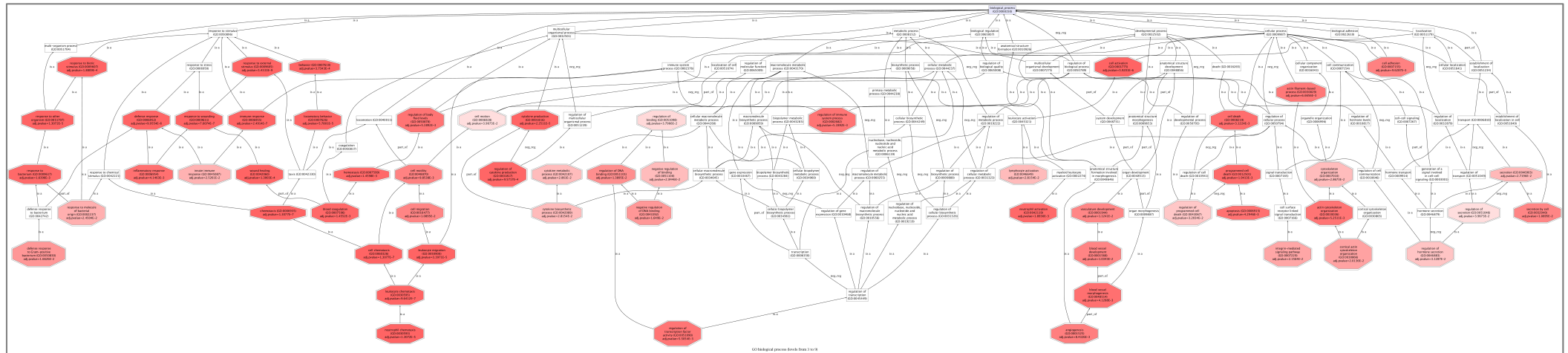

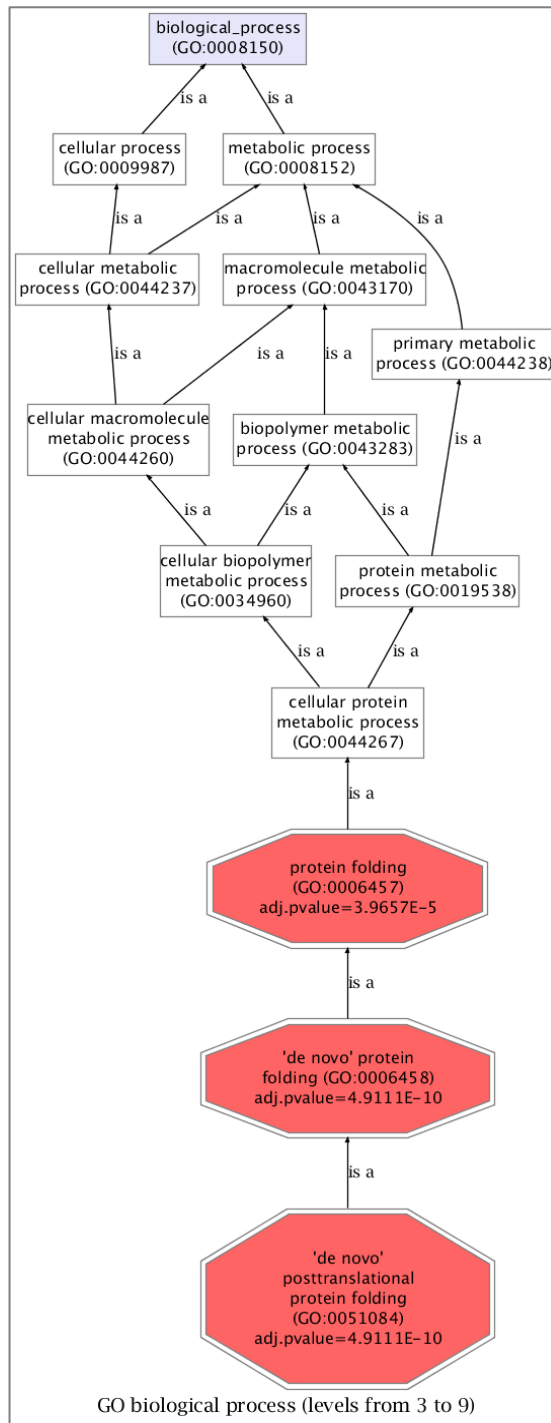

CPMP 12h

## ICMP 2-4-6h

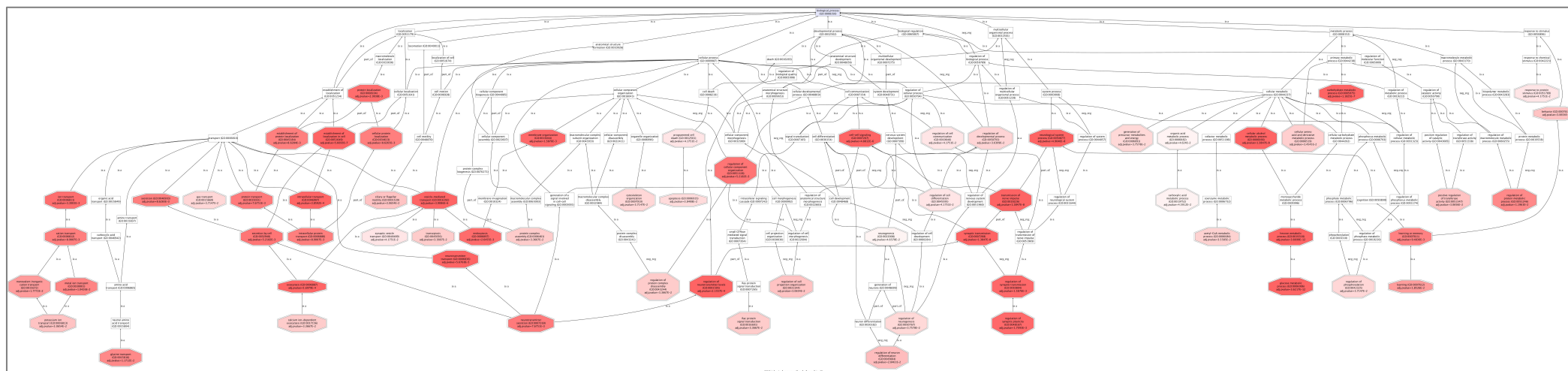

## ICMP 8h

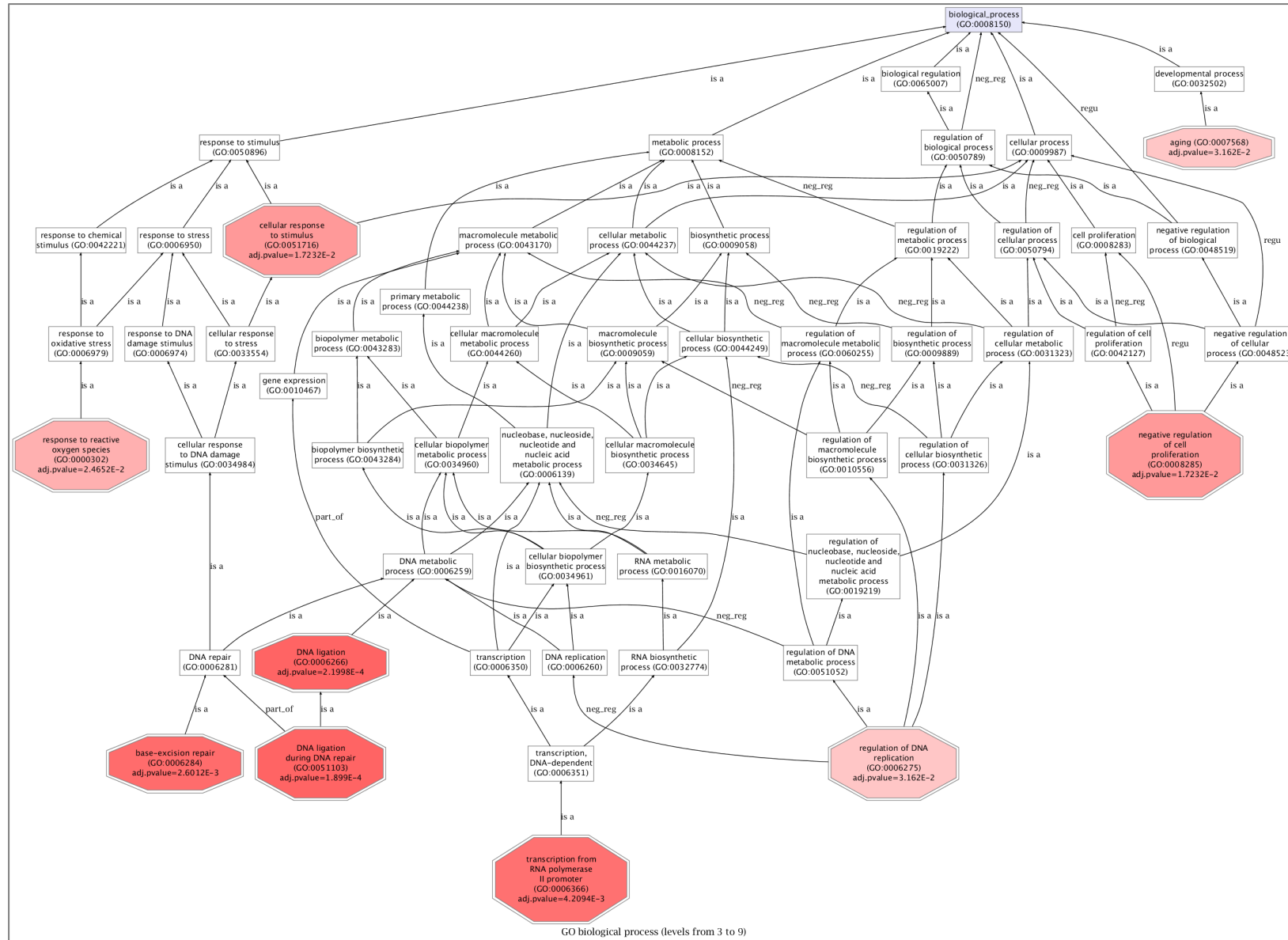

ICMP 12h

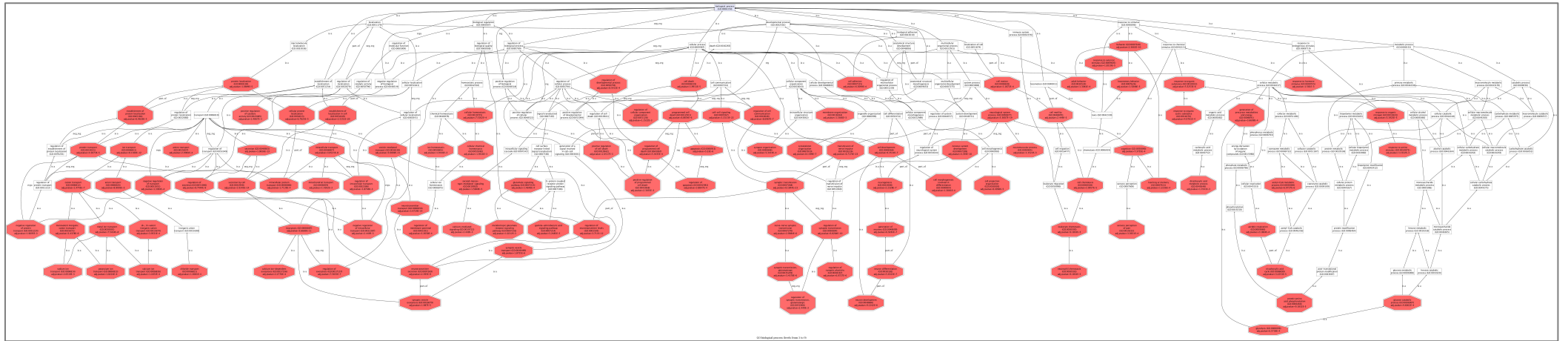

## ICMP 24-48h

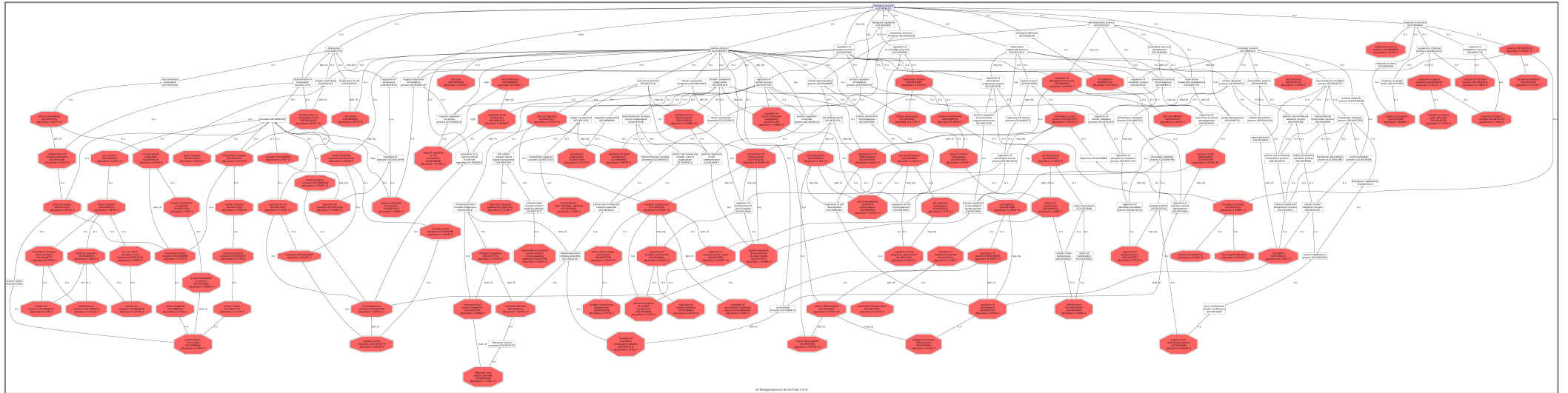

Supplement: Supplementary file 1 [file ijms-22-13024-s001.zip › Supplementary File 2.pdf]
